# Long-term aging of Ag/a-C:H:O nanocomposite coatings in air and in aqueous environment

**DOI:** 10.1088/1468-6996/16/2/025005

**Published:** 2015-03-27

**Authors:** Martin Drábik, Josef Pešička, Hynek Biederman, Dirk Hegemann

**Affiliations:** 1 Empa, Swiss Federal Laboratories for Materials Science & Technology, Laboratory for Advanced Fibers, Lerchenfeldstrasse 5, 9014 St.Gallen, Switzerland; 2 Charles University in Prague, Faculty of Mathematics and Physics, Department of Physics of Materials, Ke Karlovu 5, 121 16 Prague 2, Czech Republic; 3 Charles University in Prague, Faculty of Mathematics and Physics, Department of Macromolecular Physics, V Holešovičkách 2, 180 00 Prague 8, Czech Republic

**Keywords:** aging, nanocomposites, plasma polymerization, silver, sputtering, 10.10 nanoscale materials, 20.11 thin FILMS/nanomaterials synthesis, 30.04 materials observation (optical microscopy, TEM, SEM, SPM, SNOM), 30.07 optical analysis (IR, Raman scattering, UV), 30.10 surface/nanostructures characterization

## Abstract

Nanocomposite coatings of silver particles embedded in a plasma polymer matrix possess interesting properties depending on their microstructure. The film microstructure is affected among others also by the RF power supplied during the deposition, as shown by transmission electron microscopy. The optical properties are characterized by UV–vis–NIR spectroscopy. An anomalous optical absorption peak from the Ag nanoparticles is observed and related to the microstructure of the nanocomposite films. Furthermore, a long-term aging of the coatings is studied in-depth in ambient air and in aqueous environments. It is shown that the studied films are not entirely stable. The deposition conditions and the microstructure of the films affect the processes taking place during their aging in both environments.

## 1. Introduction

Metal/plasma polymer composite coatings attract a long-term interest in research due to a wide range of possible applications as a result of a great potential in tuning their mechanical, electrical and optical properties in various different directions according to the special requirements of any particular application [1–5]. Such films are composed of metal particles embedded in a matrix of a plasma polymer and often denoted as M/C:X, where M stands for a metal, C for carbon and X for any element present in the plasma polymer matrix (typically hydrogen, oxygen or fluorine). The term plasma polymer denotes a material that is formed as a result of an electric discharge in an organic gas, plasma-enhanced chemical vapor deposition (PE-CVD). Different to conventional polymers, a plasma polymer means a new class of material that consists of short chains that are randomly branched and terminated with a high cross-linking density. These nanocomposite films have already been identified as a suitable type of material for sensor or optical filter applications [6, 7]. Lately, composites containing nanoparticles of silver have been particularly widely studied due to the well-known antibacterial activity of silver ions [8–10]. The intention has been to use them in biomedicine as a wound dressing or in bone cements [11]. The use of silver nanoparticles is nowadays widespread in medical use and also in our everyday lives. However, not all the questions regarding the toxicity of so called ‘nanosilver’ (and nanoparticles generally) in the human body and in nature have been so far properly answered [12]. Especially, there are concerns about the Ag nanoparticles (Ag^0^) themselves, as well as the long-term exposure to Ag^+^ ions which leach from the particles in the presence of water [13, 14]. It has been shown that the Ag^+^ ion release occurs not only from free Ag particles dispersed in water but also from particles bound within a polymer matrix. In this case, the Ag^+^ release is influenced by the Ag^0^ content, as well as by the nanoparticle morphology and distribution [15]. Generally, the amount of metal determines the properties of such a composite material. When the amount of metal in the film is low, metal nanoparticles are separated from each other in the plasma polymer matrix and the coatings behave mostly as electrical insulators. On the other hand, the separation vanishes with a high amount of metal inclusions and the character of the films resembles conductors. The gradient intermediate phase between these two limiting structural regimes is called the percolation threshold. The structure of a metal/plasma polymer nanocomposite coating is typically described by a so-called ‘filling factor’ *f* (volume fraction ratio). It is defined as the volume of metal inclusions embedded in a plasma polymer matrix in the total volume of the composite material.

A commercial application of metal/plasma polymer films might require either long-term stability of the coating or its controlled aging over the period of application, e.g. the mentioned metal ion release [15], or matrix degradation [16]. The concerns about potential instability of the structure of metal/plasma polymer coatings can be considered as one of the main reasons why these materials have failed to be wide-spread in industrial applications requiring stable coatings [17]. Both the plasma polymer matrix and the metallic component of the composite are prone to aging. The plasma polymer matrix typically undergoes several structural changes when exposed to the ambient atmosphere after deposition due to the presence of free radicals, structural defects or residual stress [18]. Oxidation of a hydrocarbon matrix is among the most common processes. Reviews by Hlidek *et al* give a summary of various oxidation processes taking place in Ag-containing nanocomposite films which are also important for the initiation of Ag^+^ release [19, 20]. Particularly, oxygen and water vapor were shown to be the most active oxidative agents. The embedded metal nanoparticles are often affected by changes in their size and shape distributions. There were several mechanisms of their restructuring described: atomic diffusion along the particle surface (recrystallization and coalescence) and through the matrix (Ostwald ripening) and particle migration [1, 21]. All of these might be further enhanced by thermal activation during high temperature treatment or by laser or electron irradiation. On the other hand, controlled aging of a composite coating requires proper selection of metal and matrix materials and precise tuning of their structure. This is of a particular importance in the potential biomedical applications of antibacterial silver-containing composites with controlled Ag^+^ ion release [15, 22]. The various processes taking place in water and aqueous media are rather complex. The initiation of the Ag^+^ ion release requires a Ag^0^ particle to be oxidized [23, 24]. The oxidation process in turn requires the presence of both dissolved oxygen and protons. It produces reactive oxygen intermediates and might even proceed to a complete reactive dissolution under some conditions and disappearance of the solid nanoparticle phase [25]. The essential mechanism pathway can be written as follows:
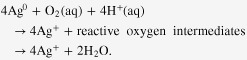



The reaction of a silver nanoparticle with H_2_O_2_ is even faster than with O_2_ so the initial nanoparticle oxidation via reaction with oxygen is the rate limiting factor. Rates of Ag^+^ ion release increase with temperature and decrease with increasing pH. The value of pH can be further influenced by the potential degradation of the plasma polymer and its resulting products. The released Ag^+^ ions can readsorb on silver nanoparticle surfaces (and be reduced by them, i.e. Ostwald ripening in the case of plasma polymer nanocomposites), so even simple colloids contain at least three forms of silver: solids (Ag^0^), free Ag^+^ ions or their soluble complexes and surface-adsorbed Ag^+^ with the possible occurrence of Ag oxides.

Overall, the tendency to nanostructural changes (their rate and overall measure) is to a great extent determined by the matrix material, its chemical structure, stability, glass transition and crosslinking. Especially, the wettability and ability of the matrix to uptake water is crucial for both the aging of the polymer (possible hydrolysis of the backbone chain) as well as for the structural modifications of the metal nanoparticles. The silver ion release was found to increase exponentially with the maximum water absorption by the polymer matrix and the diffusion coefficient of water within the matrix [22, 26]. Further, the Ag^+^ release rate decreases with increasing crosslinking of the matrix [27], and also its efficiency is higher in hydrophilic polymers compared to hydrophobic [28]. An increasing crystallinity of the matrix was found to decrease the Ag^+^ release [29]. All of these parameters affect the diffusion coefficients of water molecules as well as the Ag^+^ ions through the polymer matrix. In the case of the plasma polymer nanocomposites, the changes in the structure and properties of the silver-containing coatings during aging in air or in water and have been previously studied both on amorphous hydrophobic matrices (hydrocarbon, Ag/a-C:H, or fluorocarbon, Ag/a-C:F) [30–34], as well as on hydrophilic matrices (oxygenated hydrocarbon, Ag/a-C:H:O, or organosilicon, Ag/a-C:Si:O) [15, 35]. Apart of the matrix material, also the overall film architecture plays an important role. For example, plasma polymer multilayer films with metal nanoparticles buried in between typically show a good long-term stability [1], particularly for Au/C:F systems [36].

In this contribution, we present a detailed study of structure and optical properties of Ag/a-C:H:O nanocomposite coatings with a broad range of microstructures around the percolation threshold. The aging (in air or in water) of different a-C:H:O plasma polymer films has recently been investigated [16, 37, 38]. A rather stable plasma polymer matrix deposited from CO_2_/C_2_H_4_ discharges has thus been selected. Therefore, this work particularly focuses on the long-term aging of Ag nanoparticles embedded in such plasma polymer films in ambient air and on the influence of an aqueous environment on their structure and optical properties. Finally, a particular suitable application is suggested for each type of nanocomposite structure.

## 2. Experimental section

### 2.1. Deposition of Ag/a-C:H:O coatings

The studied nanocomposite coatings of silver particles embedded in a hydrophilic amorphous oxygenated hydrocarbon plasma polymer matrix (Ag/a-C:H:O) were deposited by a combination of PE-CVD and physical vapor deposition processes within a low pressure glow discharge. A plane-parallel capacitively coupled plasma reactor (∅ = 30 cm × *L* = 5 cm) was used. Ag electrode (∅ = 13 cm) was mounted on the top wall inside the plasma reactor and insulated by a ceramic plate. The Ag sputtering target (intrinsic purity of 99.9 vol%) was connected to an RF power generator (13.56 MHz, Cesar 133, Advanced Energy, USA) through a matchbox. The smaller diameter of the driven electrode, as compared to the grounded base plate, yielded strong asymmetrical conditions and built up a high (negative) bias voltage. The chamber and the silver sputtering target were mechanically cleaned prior to each experiment to remove all by-products of the plasma deposition process and thus maintain the same well-defined starting experimental conditions. The chamber was pumped through the bottom electrode (grid) using a combination of roots and rotary vane pumps. More details on the experimental chamber can be found elsewhere [15]. Working gases were introduced into the chamber through a gas shower system in the top chamber wall which enabled homogeneous plasma conditions. Argon was used for sputtering of Ag, while ethylene (C_2_H_4_) was the source hydrocarbon monomer gas for the plasma polymer matrix. In addition, a reactive carbon dioxide (CO_2_) was added to the working gas mixture resulting in oxidation processes in the gas phase and incorporation of oxygen-functional groups within a crosslinked hydrocarbon plasma polymer matrix (C:H:O) [15, 39]. All the gases were used as obtained from Carbagas, Switzerland, without any further purification (intrinsic purity of 99.99 vol%). The gas flow rates (in standard cubic centimeters per minute (sccm)) were measured by mass flow controllers (MKS, Germany). The working gas flow rate ratio Ar:CO_2_:C_2_H_4_ and the total gas pressure were held constant in all of the deposition processes at 50:6:1 sccm and 5 Pa, respectively. The gas mixture was previously found to influence the degree of the functionality of the coatings [40]. Similarly, the silver content in the composite increases with the increasing CO_2_:C_2_H_4_ ratio due to the reduced deposition rates of the plasma polymer matrix (at a constant power). Finally, the RF power input was varied between 20 and 100 W to obtain nanocomposite films with different filling factors.

The composition and stability of the plasma discharge was monitored *in situ* during every deposition process by optical emission spectroscopy (OES) using an AvaSpec-ULS2048-USB2 spectrometer (Avantes, Netherlands). The spectrometer was connected to the chamber through an optical fiber. The emission spectra were recorded in the wavelength range of 300–820 nm. The resolution of the spectrometer set-up was 0.4 nm. The obtained data was analyzed using the atomic spectra database provided by the National Institute of Standards and Technology (NIST) [41], and other literature [2, 42].

The Ag/a-C:H:O nanocomposite films were deposited on various substrates as required by different analytical techniques for characterization of the properties of the films. They were all placed on the grounded bottom electrode. After finishing the deposition process, the vacuum chamber was left being pumped for another 30 min before opening it to atmosphere to de-activate the radicals trapped in the plasma polymer matrix. This leads to substantially slower aging process in air [43]. All the studied nanocomposite films were of thicknesses (21 ± 2) nm as measured by a surface profiler Dektak 150 (Veeco, USA). The values of thicknesses were averaged at least from seven different positions on the glass substrates.

### 2.2. Microstructure of Ag/a-C:H:O coatings

The structure of the Ag/a-C:H:O nanocomposite films deposited on carbon foils supported by Cu grids S160 (Plano, Germany) was studied by transmission electron microscopy (TEM, Jeol 2000FX, 200 kV, Jeol, Japan). The obtained micrographs were used to characterize the size and the shape of the silver nanoparticle inclusions and their distributions. The obtained digital micrographs were corrected for contrast and/or brightness where necessary. Images were then statistically processed by ImageJ v.1.43q software [44]. The size of the embedded Ag nanoparticles was described in terms of ‘equivalent nanoparticle diameter’, *d*, defined as the diameter of an equivalent circular area. The measurement error of *d* can be estimated to be 3 pixels which corresponds to about 1 nm at the magnification of 200 000× used for analysis. The dispersion of the nanoparticle sizes was expressed in terms of the standard deviations of the size distributions. Further, ‘shape factor’ *S* of the projections of the Ag nanoparticles was studied. It is often called ‘circularity’ and defined as 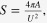 where *A* is the area of the particle projection as observed by TEM and *U* is the particle circumference. It is used to measure how close a projection of a particle is to a perfect circle. The values for the particle shape factor lie in the range 0 ≤ *S* ≤ 1, where *S* = 1 corresponds to a perfect circle [1]. Nanoparticle size and shape distributions were obtained from statistical processing of sets of several micrographs (magnification 200 000×). The size distributions were fitted by logarithmic normal (log-normal) distribution functions *f*_LN_(*d*) from which modal values of diameters *d*_m_ were obtained. Further, the crystal structure of the embedded silver nanoparticles was estimated from electron diffraction patterns obtained by selected-area diffraction (SAD) analysis according to the database provided by the International Centre for Diffraction Data (ICDD) [45].

### 2.3. Optical properties of Ag/a-C:H:O coatings

The optical properties of the Ag/a-C:H:O nanocomposite films deposited on microscopic glass slides were characterized by ultraviolet–visible–near-infrared (UV–vis–NIR) spectrophotometer Lambda 9 (Perkin-Elmer, USA) in the spectral range 300–2500 nm. The optical properties of the nanocomposites were characterized on samples deposited in the same deposition process as the corresponding films for the characterization of the microstructure by TEM. The transmittance spectra were collected using an integration sphere and certified reflectance standard Spectralon USRS-99-010 (SphereOptics, Germany) as a light spectral reference. The obtained spectra were smoothed for noise removal around the wavelength of 830 nm which is the position of the detector change. The UV–vis–NIR spectra are represented in terms of absorbance, which was calculated according to the relation between transmittance *T* and absorbance *A*: 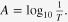 Absolute measurement error of the characterization of optical properties was estimated to be 2%.

### 2.4. Aging of Ag/a-C:H:O coatings

Aging of Ag/a-C:H:O coatings was studied in ambient air and in distilled water. In both cases, the films were left to age for a given time in the dark at a room temperature of 20 °C. In the case of measurement by TEM, the films were characterized right after the deposition (as-prepared) and after 2, 10, 18 and 23 months from the deposition for aging in air and after 1 h and 1 day in distilled water. Further, the structure of the films stored in water for 1 day was also characterized by TEM after 2 months. In the case of the measurement of the optical properties, the films were characterized right after the deposition (as-prepared) and after 1, 5, 9 and 22 months of aging in air and after 1 h and 1 day of aging in distilled water.

## 3. Results and discussion

### 3.1. Control of the deposition process

**Figure 1.**
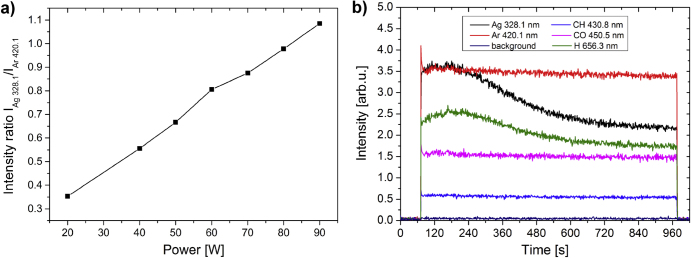
Ratio of intensities of emission lines of Ag (328.1 nm) and Ar (420.1 nm) as recorded during sputtering of Ag in Ar/CO_2_/C_2_H_4_ atmosphere (gas flow rates of 50/6/1 sccm, respectively) at a pressure of 5 Pa and various RF powers (a). Time evolution of selected emission lines during deposition at 50 W (b).

The composition and stability of the plasma discharge during the deposition of Ag/a-C:H:O nanocomposite films was controlled *in situ* using OES. An example of a typical spectrum with identification of the emitting species can be found elsewhere [7]. Major emitting species comprise Ag, Ar and H atoms and CO and CH radicals. Especially important are the atomic emission lines of Ag species (^2^P_3/2_ and ^2^P_1/2_) originating from excited states after sputtering from the metal target that can be found at 328.1 nm and at 338.3 nm, respectively. The intensity of the emission line of Ag is proportional to the sputtering rate and can thus be related to the amount of the deposited metal in the resulting nanocomposite [31, 46]. As can be seen in figure 1(a), the ratio of the intensities of Ag and Ar emission lines increases linearly with the increasing RF power of the discharge. The ratio was calculated from values of the intensities of the selected emission lines averaged over the first minute of each of the depositions. The reasons for this will be explained further. Monitoring the level of intensity of one of the emission lines of silver is a convenient means for controlling the deposition rate of silver, which is a factor determining the final filling factor of the deposited nanocomposite film. A fine control of the silver sputtering rate is important for the preparation of films with repeatable quality and composition.

The stability of the deposition process was controlled through monitoring of intensities of emission lines of several important species. An example of a time evolution of emission lines of Ag (328.1 nm), Ar (420.1 nm), H (656.3 nm), CH (430.8 nm) and CO (450.5 nm) during the deposition process at 50 W can be seen in figure 1(b). As can be seen, the emission lines of Ar, CH and CO species are almost constant throughout the whole deposition process which reflects the stable energetic and gas composition conditions. On the other hand, we can observe a strong decrease in the intensities of Ag and H emission lines throughout the deposition process. The decrease in the intensity of the Ag signal reflects the decrease of the Ag deposition rate. This is the result of an effect commonly known as ‘target poisoning’ that occurs during sputtering in an atmosphere composed of reactive gases [47]. The fragments of the reactive gases react with the surface of the target and continuously passivate it which in result lowers the metal sputter yield. Note, however, that the embedded Ag nanoparticles in the growing film are metallic despite using oxidative plasma [15]. It was shown that silver oxide (Ag_2_O) can only be produced in a pure oxygen plasma at very low growth rates (∼0.3 nm min^−1^) [48], while the growth rate for Ag was well above 1 nm min^−1^ for all conditions as used in this work (with a less oxidative plasma at the same time). Nevertheless, the target poisoning leads to a vertically inhomogeneous structure of the nanocomposite film. A typical vertical gradient in the amount of metal in the nanocomposite films can be seen on cross-sectional TEM micrographs previously published by Körner *et al* [15]. We did not compensate anyhow for this gradual decrease of sputtering rate of Ag in our experiments. As a result, a kind of barrier layer with a lower amount of metal was formed on the top of the anticipated nanocomposite film. The advantage of these gradients lies mainly in the lowered initial burst ion release in aqueous environments as was previously reported by Körner *et al* [15]. Furthermore, these structures avoid loss of nanoparticles supporting the formation of stable nanocomposites and serve as reservoirs of silver ions. In any case, the metallic target was cleaned before each experiment so that the starting conditions were the same in each deposition (i.e. ‘metal’ sputtering mode).

Due to the vertical inhomogeneity of the studied nanocomposite films which are about 20 nm thick, we did not calculate exact values of their filling factors *f* although it is the basic parameter characterizing a metal/plasma polymer composite. However, *f* is calculated as an average value. As such, it is suitable only for a homogeneous structure and in our case might be misleading. Thus, all the properties of the films will be further discussed in the dependence on the particular RF power supplied during their deposition. Nevertheless, we will refer to films with ‘higher’ or ‘lower’ filling factors where appropriate. The atomic ratio of Ag in Ag/a-C:H:O films deposited at various RF powers was reported previously [7].

### 3.2. Microstructure of Ag/a-C:H:O films

#### 3.2.1. As-prepared films

**Figure 2.**
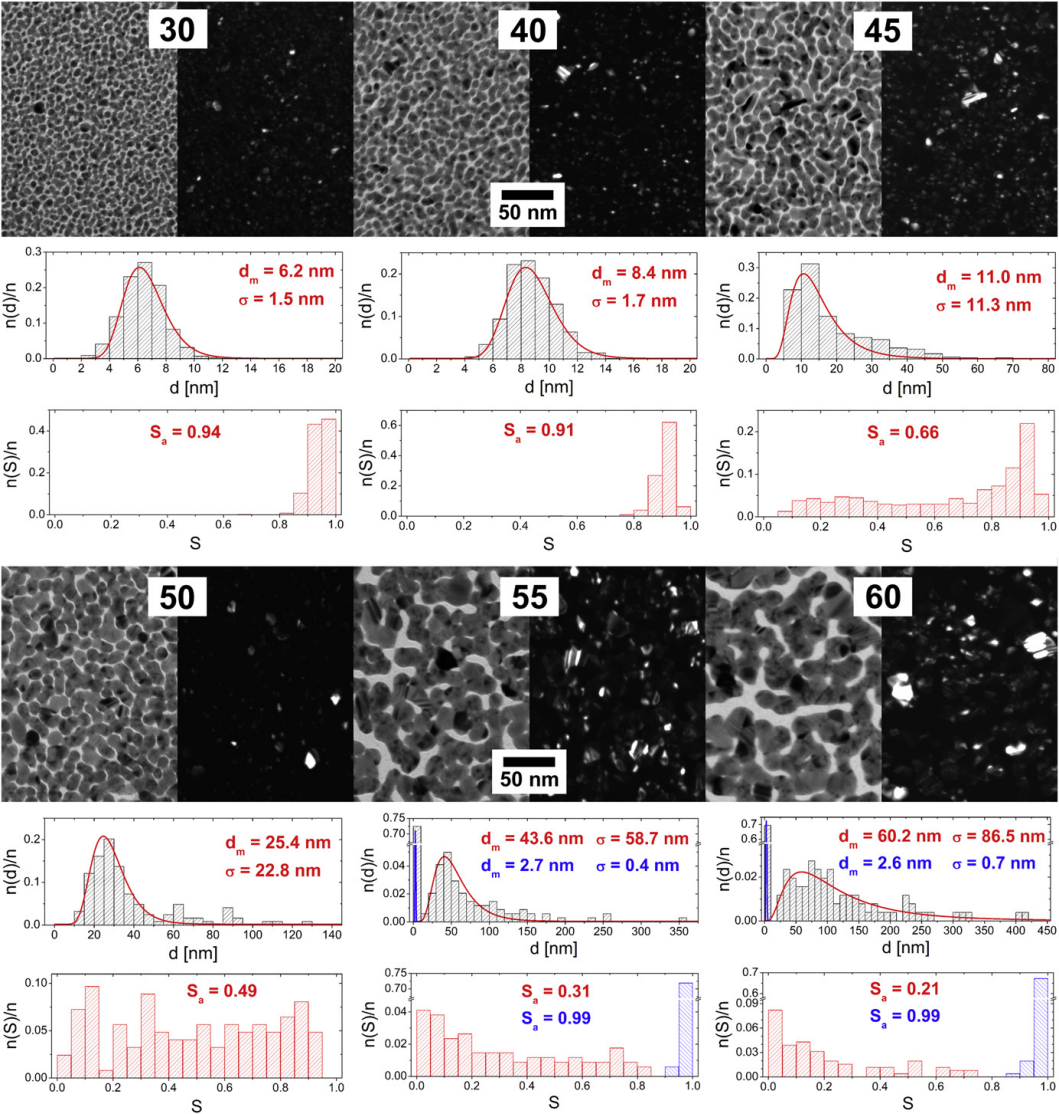
TEM micrographs of Ag/a-C:H:O nanocomposite films deposited at different RF powers (W) as measured right after their deposition. In each case, a bright field image (left) is displayed together with the corresponding dark field image of the same spot (right). For each micrograph, a distribution histogram of equivalent nanoparticle diameters *d* with its log-normal fit and modal value of nanoparticle diameter (*d*_m_) and its standard deviation (*σ*) are displayed (top). The corresponding histogram of nanoparticle shape factor *S* and the average value of shape factor (*S*_a_) are displayed (bottom).

The microstructure of Ag/a-C:H:O nanocomposite films was studied on films deposited at various RF powers delivered to the silver target. As could be expected, a broad range of powers supplied leads to nanocomposite films with considerably different microstructures. Typical TEM micrographs of selected Ag/a-C:H:O nanocomposite films deposited at various powers as measured after the deposition can be seen in figure 2. All of the films are formed of island-like structures where the silver nanoparticles are embedded in the plasma polymer matrix. In the case of the bright field images (the left-hand side micrograph for each power), the silver nanoparticles are displayed as the dark gray regions and the plasma polymer matrix surrounding them is represented by the light gray space. The nucleation and growth of metal-polymer nanostructures is governed by surface diffusion coefficient of metal atoms [49]. The size of the silver inclusions increases, while the average value of the shape factor *S*_a_ decreases with the increasing total amount of metal in the films (corresponds to an increasing power supplied). At the same, both distributions become broader. As can be seen, small (*d*_m_ = 6.2 nm) and almost circular individual silver nanoparticles homogeneously distributed within the plasma polymer are formed at the low power of 30 W, i.e. below percolation threshold. Their size increases with the increasing power. The particles start to touch their closest neighbors and coalesce into small islands (*d*_m_ = 8.4 nm) already at the power of 40 W. This can be seen also on the decreasing shape factor, although the islands are still convex. Nevertheless, the particles are still to a great extent separated by the plasma polymer matrix. As the power slightly increases to 45 W, the formed islands grow further (*d*_m_ = 11.0 nm) and start to be more and more interconnected, i.e. reach percolation threshold. At this point, structures of almost any shape can be find in the film (0.05 < *S* ≤ 1). Even though most of the particles are still almost circular, some concave island can be found. The depositions at further increased powers lead to formations of larger metal inclusions in the composite and its structure is above the percolation threshold. The modal value of the equivalent nanoparticle diameter *d*_m_ of the nanoparticles in the nanocomposite films deposited at 60 W was estimated to be 60.2 nm. At the same time, the structure of the islands becomes more and more irregular and the shape factor gradually decreases down to about 0.1. However, it is interesting to note that the depositions at 55 and 60 W lead (apart from larger nanoparticles) also to the formation of larger ‘interstitial space’, or gaps, where the separation of the island-like structures is larger than can be observed at the lower deposition powers. After a closer investigation and magnification of the micrographs, it can be seen that the plasma polymer matrix in this interstitial space contains a considerable number of very small, almost circular Ag nanoparticles with *d*_m_ ∼ 3 nm. They are represented with the blue color in the histograms of equivalent nanoparticle diameters and shape factors in figure 2. These nanoparticles are most probably located close to the film–substrate interface where they were deposited in the early stages of film growth [15]. Their further growth into large particles and/or islands was hindered by the growing plasma polymer matrix. These nanoparticles will be further discussed in the following sections.

The dark field images are displayed at the right-hand side for each power. In this case, the diffracting crystals are represented by the bright spots on the dark background. The images reveal the monocrystalline structure of the individual silver nanoparticles. The islands merged from monocrystalline nanoparticles of various sizes and crystal orientations grown at higher powers are typically multicrystalline.

#### 3.2.2. Aging in ambient air

**Figure 3.**
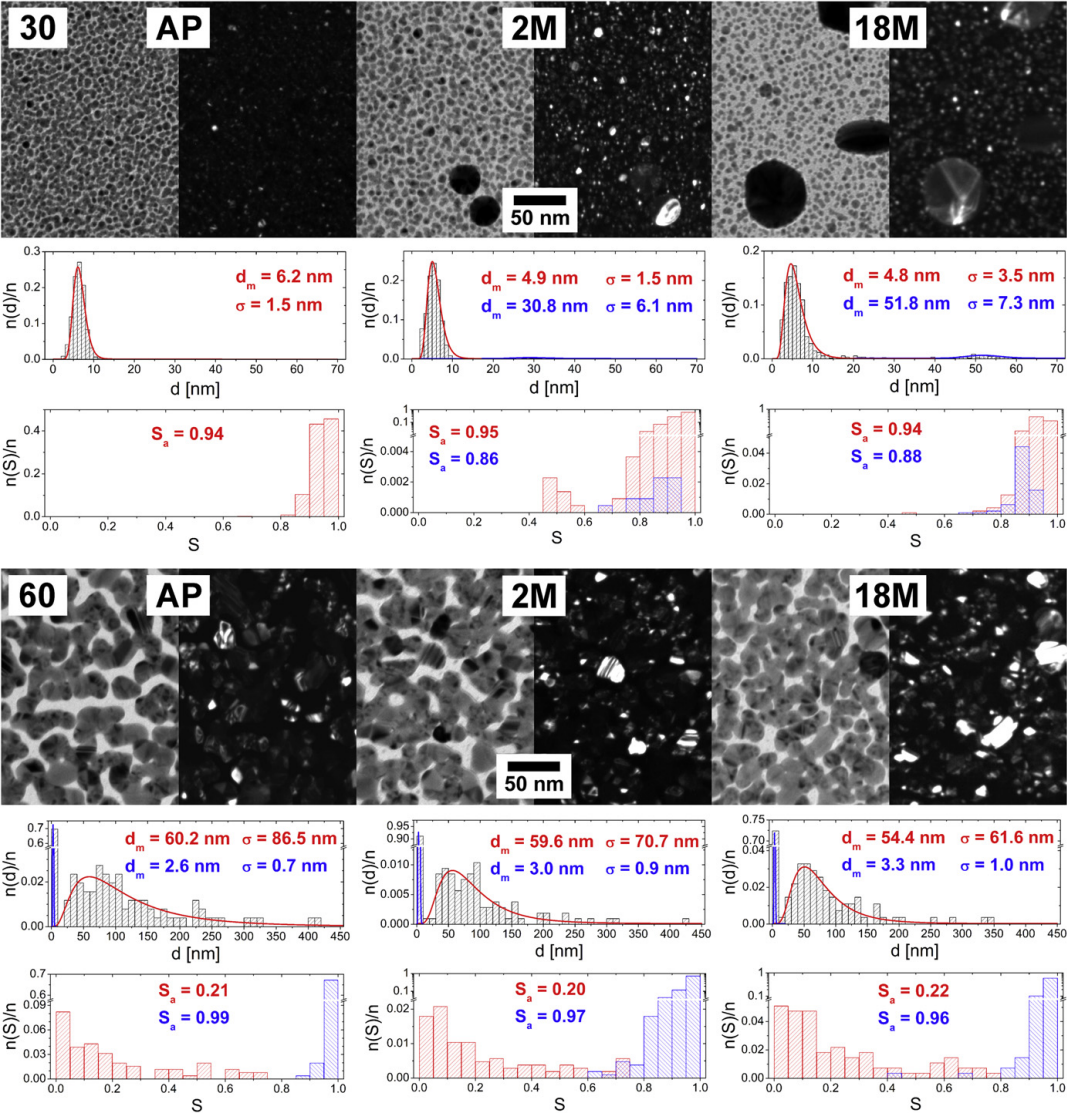
TEM micrographs of Ag/a-C:H:O nanocomposite films deposited at RF power of 30 W (top) and 60 W (bottom) as measured right after the deposition (left), 2 months after the deposition (middle) and 18 months after the deposition (right). In each case, a bright field image (left) is displayed together with the corresponding dark field image of the same spot (right). For each micrograph, a distribution histogram of equivalent nanoparticle diameters *d* with its log-normal fit and modal value of nanoparticle diameter (*d*_m_) and its standard deviation (*σ*) are displayed (top). The corresponding histogram of nanoparticle shape factor *S* and the average value of shape factor (*S*_a_) are displayed (bottom).

The characterization of microstructure of the Ag/a-C:H:O nanocomposite films was repeated after the samples aged in ambient air for several days and months after the deposition. TEM micrographs of nanocomposite films deposited at powers of 30 and 60 W, as observed right after the deposition, and 2 and 18 months from the deposition can be seen in figure 3. These two films were selected because they represent the two limiting cases in our study: from the point of view of the discharge power (below and above the percolation threshold), as well as the behavior during aging. All the other characterized films will be discussed further below.

The changes in the structure of the films deposited at the lower power of 30 W that take place during the aging of the films in air are obvious at first sight: a gradual growth of several nanoparticles into larger objects at the expense of the other small nanoparticles which serve as the source material for their growth. The modal value of the equivalent nanoparticle diameter *d*_m_ of the intrinsic small nanoparticles slightly decreases from about 6 nm down to about 5 nm, while *d*_m_ of the grown large crystals (marked with blue color in the graphs) increases to about 31 and 52 nm after 2 months and 18 months of aging in air, respectively. It is important to note that the projections of all the observed nanoparticles remain mostly circular. However, the images of the dark field and the SAD analysis discussed further below suggest that all the particles are monocrystals. Their growth most probably proceeds through atom/ion diffusion (Ostwald ripening) rather than a particle migration and coalescence. Ostwald ripening and the atom/ion diffusion through the matrix are driven by the concentration gradient around particles of different size which is the kinetics factor for the diffusion. Particularly, the concentration next to larger particles is lower than around those smaller and in result, larger particles grow at the expense of smaller ones. The growth of larger particles is further affected by minimization of the surface energy, where the larger particles also become more energetically stable [1]. The water vapor from the ambient air penetrating the hydrophilic C:H:O plasma polymer matrix most probably supports this structural change by enabling the Ag^+^ ion release from the silver nanoparticles and even enhancing the ion mobility. Since the nanocomposite is not in a direct contact with water, the silver ions are not lost and rather contribute to the growth of some of the nanoparticles. It is interesting to note that while the size of the small intrinsic nanoparticles remains almost the same in time, their number obviously decreases. Since their modal diameter is about 6 nm and their size distribution relatively narrow (no particles larger than 10 nm were observed) and the film thickness is about 20 nm, it can be expected that most of the nanoparticles are fully embedded within the plasma polymer matrix. Further, it can be assumed that the changes occur at first with the nanoparticles which are at or close to the film surface and only afterwards proceed deeper in the film [50]. Therefore, the nanoparticles close to the film–substrate interface remain untapped within the first two years of aging in air. It also important to mention that the small particles located at the substrate surface are better stabilized, because they are in fact only half-spheres (i.e. have smaller surface than the structures fully embedded in the plasma polymer matrix) and also the plasma polymer matrix tends to be more crosslinked in the early stages of the film growth [51]. The growth of metal nanoparticles in Ag/C:H composites, together with the increase in the nearest-neighbor distance within the first few days of their aging in air, was reported also by Biederman *et al* [30].

The Ag/a-C:H:O nanocomposite films deposited at the high power of 60 W are relatively stable in air when compared to those prepared at the lower power. All the micrographs of the aging of this film might well seem identical at first sight. Although the composites deposited at higher powers contain more silver and complex structures and therefore the particular changes in the films are difficult to spot and follow, a careful analysis of the micrographs brings more insight. Even though the small nanoparticles situated in between the islands (marked in blue in the graphs) remain almost the same with *d*_m_ of about 3 nm, a slight disruption of the percolation structure of the nanocomposite film can be observed after 18 months of aging. The nanoparticles previously fully merged in the island-like structure are in the end more sharply delineated and even individual nanoparticles can be observed at several positions. Even though the wide distribution of the shape factor remains unchanged, the disruption of the percolation structure is supported by the statistical processing of the equivalent nanoparticle diameter, where *d*_m_ decreases from 60.2 nm for the as-deposited films down to 59.6 nm after 2 months and to 54.4 nm after 18 months. This suggests that the island-like structure of the nanocomposite loses material, particularly from the ‘neck’ interconnections of the nanoparticles. The causes of the aging might be the water vapor that penetrates the hydrophilic matrix and enhances the silver release. However, it is not entirely clear what happens with the lost silver atoms when the films are stored in air. It is possible that the silver ions released from the ‘necks’ of the island-like structures are reduced on the top of the spherical nanoparticles which thus grow in the vertical direction. This is a process that cannot be detected from the TEM 2D projections and a cross-sectional analysis would be necessary.

From the micrographs in figure 3, it might seem that the aging processes taking place in the film deposited at 60 W are much less pronounced than in the case of the film deposited at the low power of 30 W. Particularly, that the film with the higher filling factor deposited at 60 W loses less silver. However, this observation might be misleading because the TEM micrographs provide only a 2D projection of the structure. No conclusion on the total release of silver can be made. On the contrary, it has already been shown that the intrinsic amount of silver influences the Ag^+^ ion release: the higher the initial silver content, the higher the total release of silver was observed [15]. Although the mentioned study was performed in water, we can expect that a similar effect might take place here due to the water vapor, even though on a smaller scale. On the other hand, the properties of the plasma polymer matrix contribute to the stability of the composite. The deposition at higher powers results in a more crosslinked plasma polymer which slows down both the penetration of water molecules and possibly also the resulting atom/ion diffusion through the matrix [22]. Also, a high surface area might greatly enhance the Ag release with respect to the total Ag^0^ content.

#### 3.2.3. Aging in distilled water

**Figure 4.**
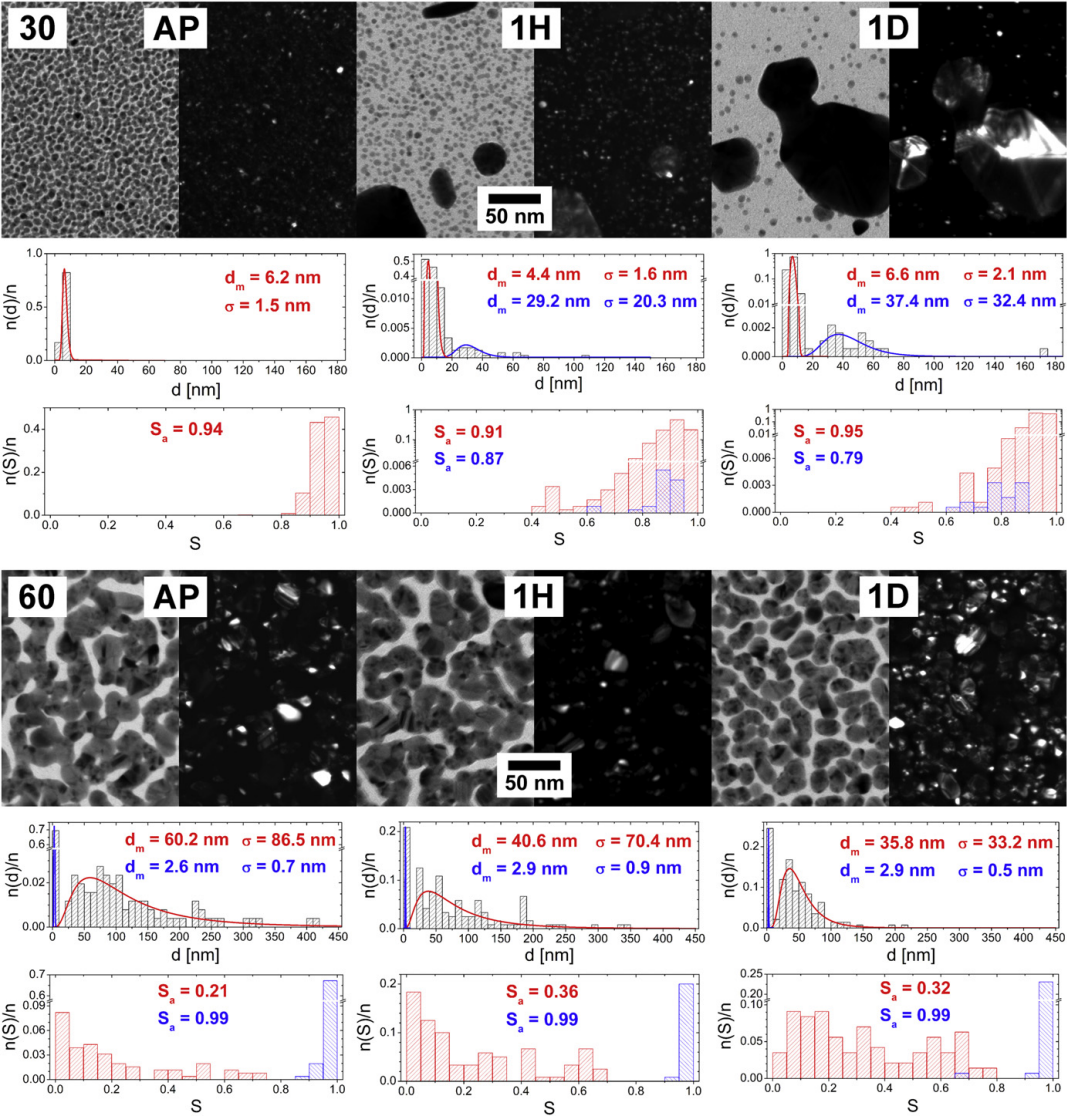
TEM micrographs of Ag/a-C:H:O nanocomposite films deposited at RF power of 30 W (top) and 60 W (bottom) as measured right after the deposition (left), after 1 h in distilled water (middle) and after 1 day in distilled water (right). In each case, a bright field image (left) is displayed together with the corresponding dark field image of the same spot (right). For each micrograph, a distribution histogram of equivalent nanoparticle diameters *d* with its log-normal fit and modal value of nanoparticle diameter (*d*_m_) and its standard deviation (*σ*) are displayed (top). The corresponding histogram of nanoparticle shape factor *S* and the average value of shape factor (*S*_a_) are displayed (bottom).

The microstructure of the Ag/a-C:H:O nanocomposite films was studied after their immersion into distilled water for 1 h and 1 day. Körner *et al* found in the previous study that most of the Ag^+^ release from similar samples with structures below the percolation threshold of a comparable thickness of 25 nm occurs within the first day of storage in the water bath [15]. The TEM characterization was repeated again two months after the samples were taken out from the water bath to control if the aqueous environment triggers any further changes in the structure of the films. TEM micrographs of nanocomposite films deposited at powers of 30 and 60 W, as observed right after the deposition, and after 1 h and 1 day of storage in distilled water are displayed in figure 4.

The soaking of the Ag/a-C:H:O film deposited at 30 W into a water bath for a few hours results in two eye-catching effects. On the one hand, it is the fast growth of large crystals and on the other the vanishing of the intrinsic small nanoparticles. While the modal value of the size of the small nanoparticles is about 6 nm, distribution of their shape factor becomes broader in time. Large, to a great extent circular, crystals with diameter of about 29 nm appear in the composite film already after 1 h in the water. Their size grows further and reaches about 37 nm after 1 day in water. Based on the SAD analysis discussed further, we cannot expect that all of the nanoparticles are monocrystals. Also, the growth of the large crystals in the later stages can be accompanied by the coalescence of contacting particles, as can be seen in figure 4 top right. It is important to stress that the films are about 20 nm thick and therefore the large crystals grown are most probably sticking out of the nanocomposite film. The crystals are thus in a direct contact with water solution of the free Ag^+^ ions. The released Ag^+^ ions can be readily adsorbed and reduced on their surfaces which enhances the growth of the particles. In the case of the Ag/a-C:H:O film deposited at 60 W, no growth of larger nanoparticles is found in the presence of water. Rather a disruption of the complex percolated structure can be observed in time: *d*_m_ decreases from the original 60.2 nm down to 40.6 nm after 1 h and even down to 35.8 nm after a whole day in water. The randomly shaped, mostly concave, metal islands separate into individual particles with more circular shape: the shape factor distribution gradually shifts to higher values. On the other hand, the small almost circular silver nanoparticles in between the island-like structure remain unchanged within the first day of aging in water. This observation supports the previously stated assumption that they are located close to the substrate and therefore are not affected in this early period of aging in water. Despite this, the film deposited at the higher power of 60 W obviously releases silver similar to the film deposited at the lower power of 30 W; it is not clear why the silver material is completely lost in the solution and does not contribute to the growth of larger particles as is observed in the case of the film with the lower filling factor.

When we compare the structural changes induced by water with the aging of the Ag/a-C:H:O composites taking place in air, we can see that both reveal the same characteristics. However, the changes are much more pronounced and occur at much shorter time scales in the aqueous environment. While the changes induced in water within the first hour are roughly equivalent to the aging for 2 months in air in the case of the film deposited at 30 W, they are almost equivalent to the aging for 18 months in the case of the film deposited at 60 W. This will be further discussed in the following section.

#### 3.2.4. Aging of Ag/a-C:H:O films

**Figure 5.**
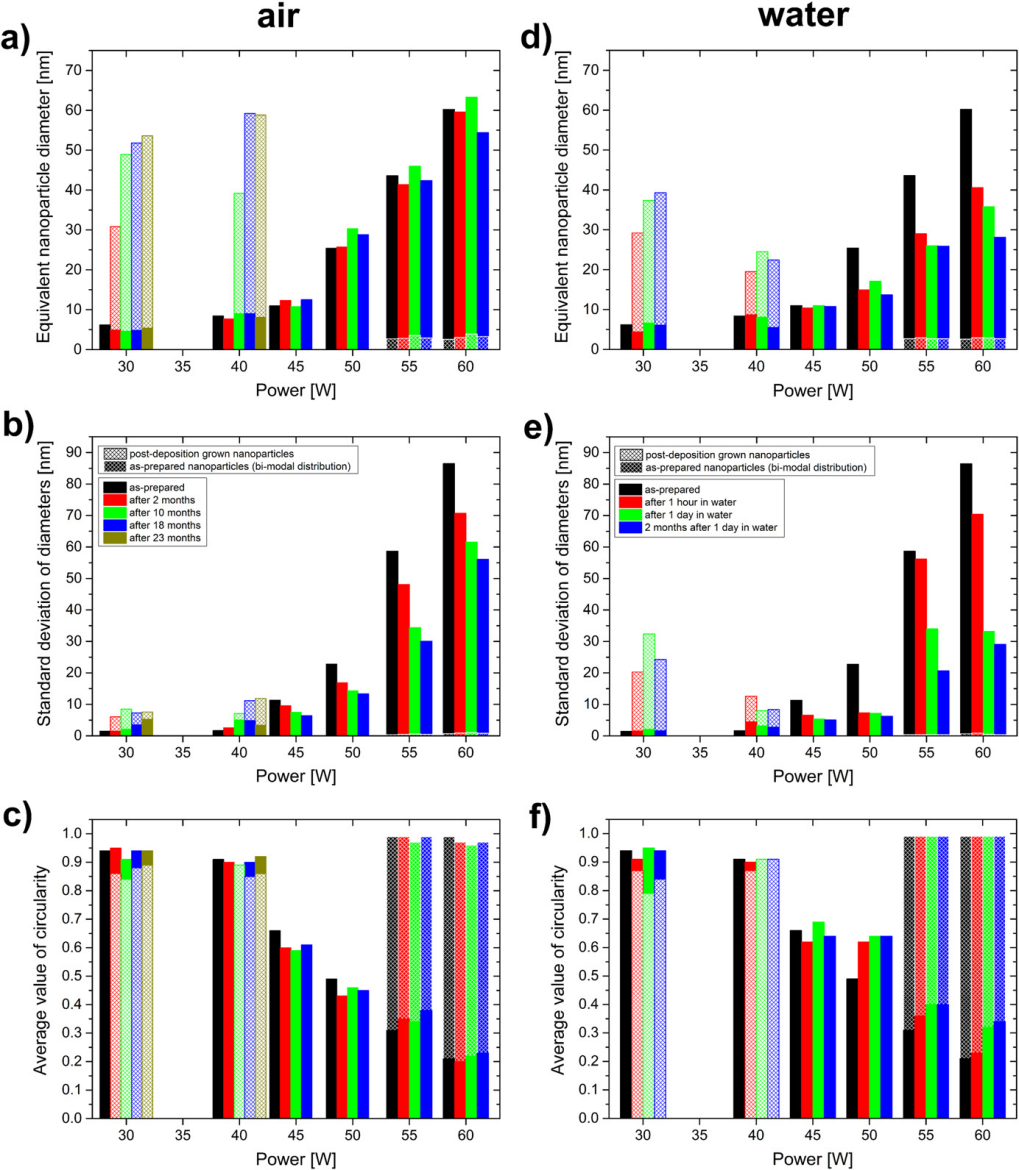
Time evolution of the microstructural parameters of Ag/a-C:H:O nanocomposite films (modal value of equivalent nanoparticle diameter *d*_m_, (a) and (d), standard deviation of diameters distribution *σ*, (b) and (e), and average value of shape factor of nanoparticles *S*_a_, (c) and (f)) deposited at different RF powers during their aging in ambient air (a)–(c) and in distilled water (d)–(f).

Apart from the Ag/a-C:H:O nanocomposite films deposited at 30 and 60 W discussed in detail above, composites with other filling factors deposited at powers between these two limiting cases were also studied. The changes in the basic microstructural parameters, i.e. modal values of the equivalent nanoparticle diameter, *d*_m_, standard deviation of distribution of diameters, *σ*, and average value of circularity of nanoparticles, *S*_a_, as recorded during the aging of the films in air or in distilled water are displayed in the dependence on the deposition power in figure 5.

As can be seen in figure 5(a), the sizes of the as-prepared metallic structures and the widths of their distributions in the Ag/a-C:H:O nanocomposites increase with the increasing deposition power, while the corresponding shape factor decreases. In the case of high powers of 55 and 60 W, we can observe a bi-modal distribution of the particle sizes: small nanoparticles distributed in between the island-like structure, as reported above. The studied films are not only of different intrinsic microstructures, also their aging in air proceeds in different directions. The most obvious changes occur with the nanocomposite films deposited at the low powers of 30 and 40 W that contain mostly individual circular silver nanoparticles separated by the plasma polymer matrix. While the size of the original as-prepared small nanoparticles does not change within about the first two years, large and almost circular particles start to grow in the films during the first months of their aging. The changes are fastest in the first months when they reach sizes of 30–50 nm. Despite this it seems that the onset of the growth of the large particles is postponed in the case of the film deposited at 40 W (no larger crystals were observed in the film after 2 months of aging in air); finally the crystals grow larger after almost 2 years (up to about 60 nm) most probably due to more silver material available for their growth. On the other hand, the nanocomposite films deposited at powers of 45 W (i.e. at the percolation threshold) and higher seem to be more stable in air. The size of the as-prepared nanoparticles is almost constant in these cases and no growth of large crystals was observed in them within 23 months of their aging in air. However, a slight decrease in the *d*_m_ of the island-like nanostructures in the film deposited at 60 W can be found after 23 months, particularly from the original size of about 60 nm down to about 55 nm. It seems that the percolated structures of the films with higher filling factors deposited at 50 and 60 W start to be disrupted, as it was discussed in detail in the section above. This process is evidenced by the narrowing size distributions and slightly increasing values of their shape factors (circularity). On the other hand, the small nanoparticles at the substrate–film interface seem to be intact during the aging of the films. Overall, it is most probable that the studied films are not in equilibrium after almost two years from the deposition and their aging in air will continue.

Figure 5(b) shows the aging in water of similar Ag/a-C:H:O nanocomposites prepared at the same experimental conditions in the dependence on the deposition power. Similarly to the aging in air, also two distinct structural changes were observed in the case of the aging in water. A fast growth of large crystals occurs in the films deposited at the low powers of 30 and 40 W already within the first hour in water, when the particles reach diameters of about 20–30 nm. The formed crystals grow further during the next one day in water. However, this growth is much slower than in the beginning, most probably due to loss of silver material into the water bath which cannot contribute to the growth. The massive leaching of Ag^+^ in water might also be the reason why the sizes of the grown metal crystals most probably do not reach the size of the crystals grown during the aging in air. There is simply not enough material for the growth of the larger nanoparticles, while its loss during the aging in air is limited and the Ag^+^ leaching induced by the air humidity promotes Ostwald ripening and recrystallization. On the other hand, we did not observe any growth of particles in water in the case of the films deposited at higher powers. Rather a degradation of the island-like structure during their aging in water is obvious. While in the case of the aging in air, the degradation was difficult to recognize and could be spotted only in the Ag/a-C:H:O nanocomposite deposited at 60 W, it is clearly visible in the TEM micrographs in figure 4 (and also evidenced by their statistical processing) of the films deposited at powers higher than 45 W after their aging in water for 1 h. The equivalent nanoparticle diameter decreases from 60.2 nm down to 40.6 nm for the film deposited at 60 W, from 43.6 to 29 nm for the 55 W film, and finally from 25.4 to 14.9 nm for the 50 W film. In correlation to the decrease in the diameter, the nanoparticle size distributions become narrower and the average values of shape factors increase. The small nanoparticles at the substrate interface are again left intact. Further, only slight changes were observed in the structure of the film deposited at 45 W. For these four films, not only the silver release is higher for the film deposited at the higher power, but also the silver release continues for a longer time. While in the case of the film deposited at 60 W, the degradation continues also during the first day in water and clearly proceeds even during its further aging in air for 2 months, in the case of the film deposited at 50 W, the degradation is very limited after the first hour in water. This observation seems to be correlation to the previous study of Körner *et al* [15], where a clear dependence of the Ag^+^ release on the initial silver content was shown: an increase in the Ag content in the coating results in an increasing Ag^+^ release in distilled water. From the TEM characterization, it is difficult to give any similar comparison of total silver released also to the two films deposited at 30 and 40 W, because at least part of the released ions contribute back to the growth of the crystals in the films. When the films were taken out from the water bath after the one day, their microstructure did not change much and it seems that all the processes that could have proceeded, already took place in the water bath. No similar changes, as those observed on the previous series of films within their first 2 months of aging in air, were found. The only difference is the case of the film deposited at the highest power of 60 W, at which we can still track the continuing disruption of its percolated structure.

To summarize this section, we can say that two aging processes were observed during aging of the Ag/a-C:H:O nanocomposites in air or in water: nanoparticle growth and disruption of the percolated structure. Overall, these are much enhanced by the direct presence of water when compared to air (time order of hours versus months). The a-C:H:O plasma polymer matrix contains various oxygen functional groups [52], and thus allows good water penetration. The water contact angle was previously measured to be between 37° and 42° depending on the silver content [40]. It is obvious that *d*_m_ of structures in the films with higher filling factor decreases much faster than in those with lower filling factors. This supports the previous measurements that the total silver release from the films with higher filling factors is much higher although visually the structures of these films look less disrupted when compared to the films with lower factors in which mostly only the large grown crystals are left in the end [15]. One of the reasons of the low measured Ag^+^ release might be the contribution of the leached silver ions to the growth of the crystals. However, it is not clear why the crystals do not grow also in the case of films with higher filling factors. Out of all the studied Ag/a-C:H:O nanocomposite films, the one deposited at the moderate power of 45 W seems to be the most stable. No changes were observed in the structure of the film during its aging in ambient air or in distilled water. This suggests that there is a good balance of concentration of Ag atoms/ions around the silver nanostructures in the films at the percolation threshold.

#### 3.2.5. Crystal structure

**Figure 6.**
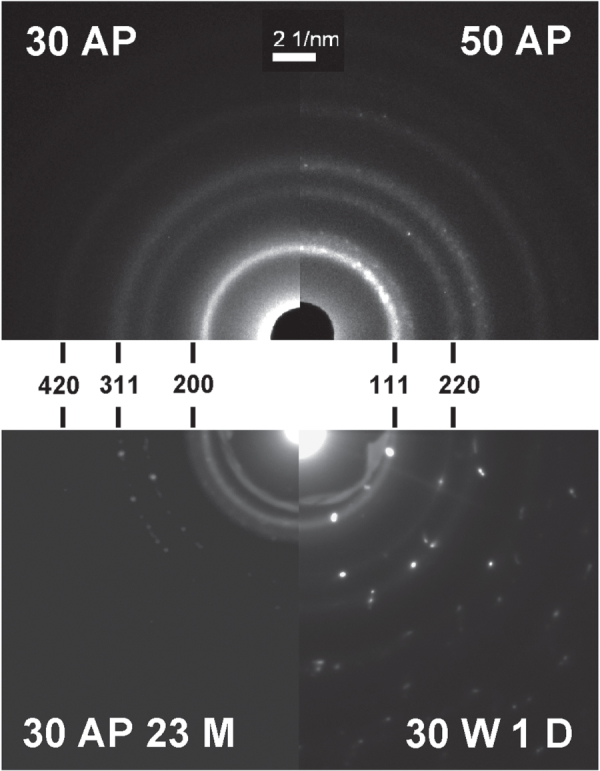
Electron diffraction patterns of Ag/a-C:H:O nanocomposite films deposited at the RF power of 30 W (top left) and 50 W (top right) as measured right after the deposition and Ag/a-C:H:O nanocomposite film deposited at RF power of 30 W, as measured after 23 months of aging in ambient air (bottom left) and after 1 day of aging in distilled water (bottom right). Each pattern is displayed with corresponding Miller indices.

The deposited Ag/a-C:H:O nanocomposite films were studied by the SAD method to reveal the nature of the crystal structure of the metallic inclusions by analysis of the electron diffraction patterns. Typical diffractograms of selected films with corresponding Miller indices are displayed in figure 6. The diffraction patterns are composed of both rings and spots. While the diffraction rings are attributed to small nanoparticles, the bright spots represent larger crystals. The SAD analysis corresponds to the conclusion from the analysis of the bright field: the size of the silver crystalline inclusions in the nanocomposites increases with the deposition power. From the analysis of the diffractograms, it can be further concluded that all the inclusions in all of the studied films are of pure Ag with the face centered cubic structure with the lattice parameter *a* = 4.086 Å, which corresponds well to the data obtained from ICDD [45]. However, the SAD analysis does not principally exclude the possibility of presence of thin oxidized layers on surfaces of the Ag nanoparticles. The growth of the nanocomposite film takes place under non-equilibrium conditions, where the deposited energy enables surface mobility and bond opening/formation. Thus, metallic Ag nanoparticles are formed within a plasma polymer matrix. In equilibrium, however, Ag metal nanoparticles get rapidly oxidized at the surface [53]. Furthermore, Ag was found to form oxygen bonds on polymers without any plasma activation and clearly more of them after an O_2_ plasma treatment [54]. From this, we can expect that the metallic Ag nanoparticles have oxidized surfaces which partly form bonds to the hydrocarbon-based plasma polymer matrix (Ag–O–C bonds).

It is interesting to note that the crystal structure of the silver nanoparticles does not change during the aging of the Ag/a-C:H:O nanocomposite films in air or in water. No signs of oxidation of the silver nanoparticles were observed. This degradation process is typically reported for the aging of Ag nanoparticles in water [55]. On the other hand, the size and the amount of the silver particles in the Ag/a-C:H:O nanocomposite film deposited at 30 W increases during its aging, while the small intrinsic nanoparticles almost disappear. Further, a detailed analysis of the aging films revealed that the large grown Ag particles can contain several crystallographic domains.

### 3.3. Optical properties of Ag/a-C:H:O films

#### 3.3.1. As-prepared films

**Figure 7.**
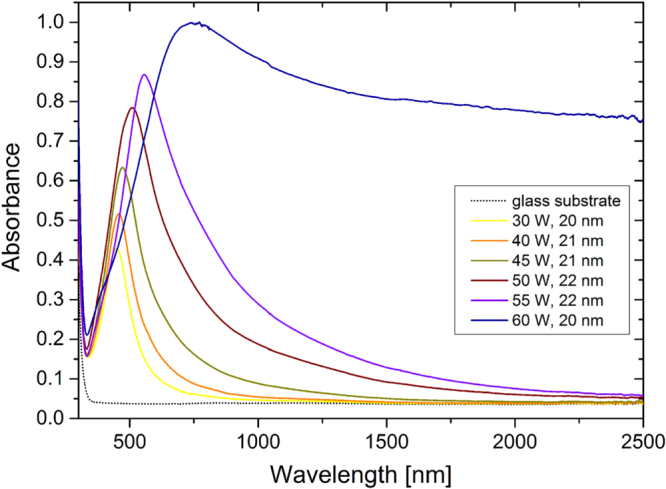
UV–vis–NIR absorbance spectra of Ag/a-C:H:O nanocomposite films deposited at different RF powers as measured right after their deposition. The optical spectrum of the glass substrate is displayed for comparison. Each of the films is listed with its corresponding thickness.

The optical properties of the as-deposited Ag/a-C:H:O nanocomposites were characterized within the UV–vis–NIR spectral ranges on samples prepared in the same deposition process as the corresponding films for the characterization of the microstructure by TEM. The absorbance spectra of the nanocomposite films deposited at different RF powers as measured right after their deposition are displayed in figure 7. As can be seen, the spectra of the Ag/a-C:H:O films deposited at powers lower than 60 W reveal a strong anomalous absorption roughly around 500 nm with a long tail of gradually decreasing absorbance towards the NIR spectral range. This absorbance peak is typical for composite nanomaterials containing metallic nanoparticles. It appears due to the well-known phenomenon of surface plasmon resonance (SPR) as a result of collective oscillations of conduction electrons after an interaction between the nanoparticle and the electromagnetic field of the light [56]. As can be seen in the spectra presented in figure 7, three characteristics of the absorbance peaks change with the different powers during the deposition: the intensity of the absorbance, the position of the absorbance maximum and also the full width at half maximum (FWHM) of the absorbance peak. Particularly, the intensity of the absorbance increases from 0.43 for the film deposited at 30 W up to 0.87 for the film deposited at 55 W, the position of the SPR gradually shifts to the longer wavelengths, from 447 nm for the film deposited at 30 W up to 557 nm for the film deposited at 55 W, and finally the FWHM increases from 133 nm for the narrowest absorption peak of the film deposited at 30 W up to 353 nm for the film deposited at 55 W. These three absorbance peak parameters are closely connected to the microstructure of the coatings as will be discussed in the following section in detail. In short, the increasing intensity of the absorption is caused mainly by the increased size of nanoparticles, while the red-shift of the peak originates in the decreasing shape factor [1, 56]. Also, the absorption peaks are much broader with the increasing deposition power due to the increasing inhomogeneity of the sizes of the silver nanoparticles (broader distributions) which gives rise to different plasmon modes [57]. Further, it can be seen that the spectrum of the nanocomposite film deposited at the power of 60 W differs from the others. The exact position of the SPR peak is difficult to locate due to the broadness of the peak and the film has rather high absorbance of about 0.8–0.9 within the whole NIR spectral range. This phenomenon can be ascribed to the absorption of light by the free electrons (inter- and intraband electron excitations) due to the percolated structure of this nanocomposite. The absorbance spectrum resembles that of a continuous silver thin film rather than the spectra of discontinuous nanocomposites with separated nanoparticles or with island-like structures revealing SPR.

#### 3.3.2. Aging of Ag/a-C:H:O films

**Figure 8.**
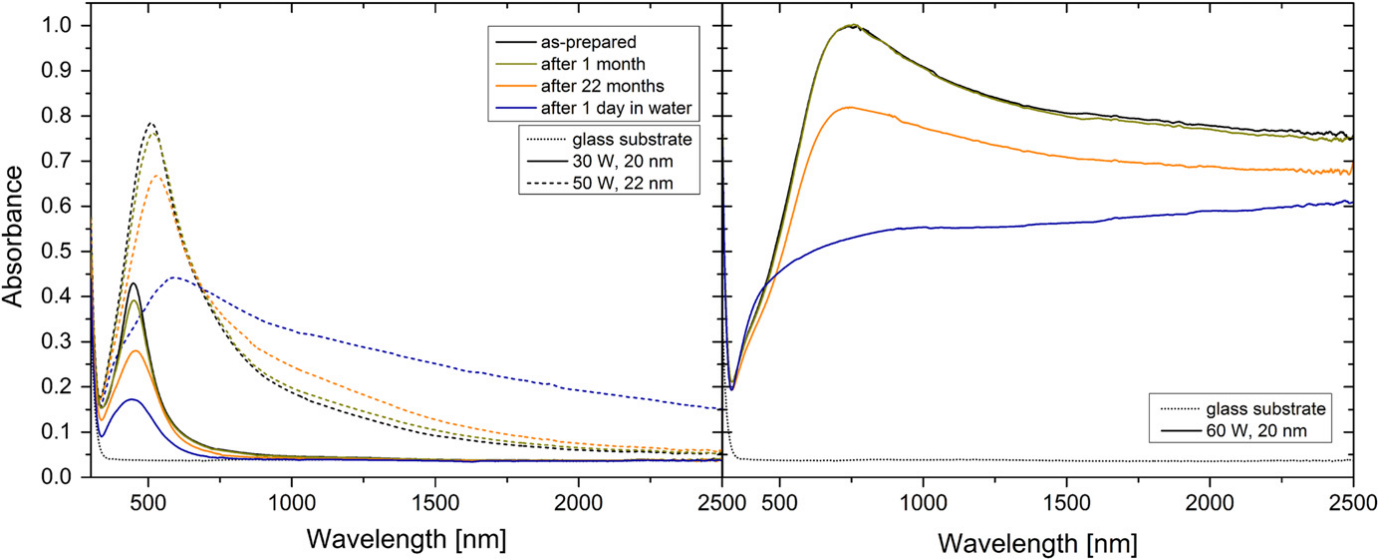
UV–vis–NIR absorbance spectra of the Ag/a-C:H:O nanocomposite films deposited at the RF powers of 30 and 50 W (left) and 60 W (right). The spectra were measured right after the deposition, and after aging in air for 1 month and 22 months and in water for 1 day. The optical spectrum of the glass substrate is displayed for comparison.

The aging of the Ag/a-C:H:O nanocomposite films in air or in water induces changes in their optical spectra as can be seen in figure 8. The dominant feature in the spectra of the aging films is the decrease in the intensity of the absorbance peak which is especially striking in the case of aging in water. This decrease is caused by the loss of silver material. However, an interesting effect can be observed in the NIR part of the spectrum of the film deposited at 50 W. While the values of absorbance of the films both with low (30–45 W) and high (60 W) filling factors decrease in this region during aging, the absorbance of the film deposited at 50 W increases. The exact cause of this effect is not known, but it might be connected with the special percolated structure of this film and the leaching of Ag^+^ ions as was discussed above. It can be expected that not all of the ions released from the silver nanoparticles also leave the matrix. They might remain (temporarily) bound within the plasma polymer and affect the permittivity of the matrix or virtually increase the percolation of the film. Since these are only distributed ions they could not be detected in the TEM characterization. Further, the positions of the SPR slightly red-shift during aging, even though, the shape factors of the films with higher filling factors increase. The maximum in the spectrum of the 60 W film aged in water cannot be identified. Finally, the FWHM measures of the SPR peaks increase during aging. Overall, the trends of the changes are the same during aging in air or in water. However, it can be seen that their progress is much faster and more pronounced during their aging in water. This observation corresponds to the observed changes in the microstructure of the nanocomposites.

**Figure 9.**
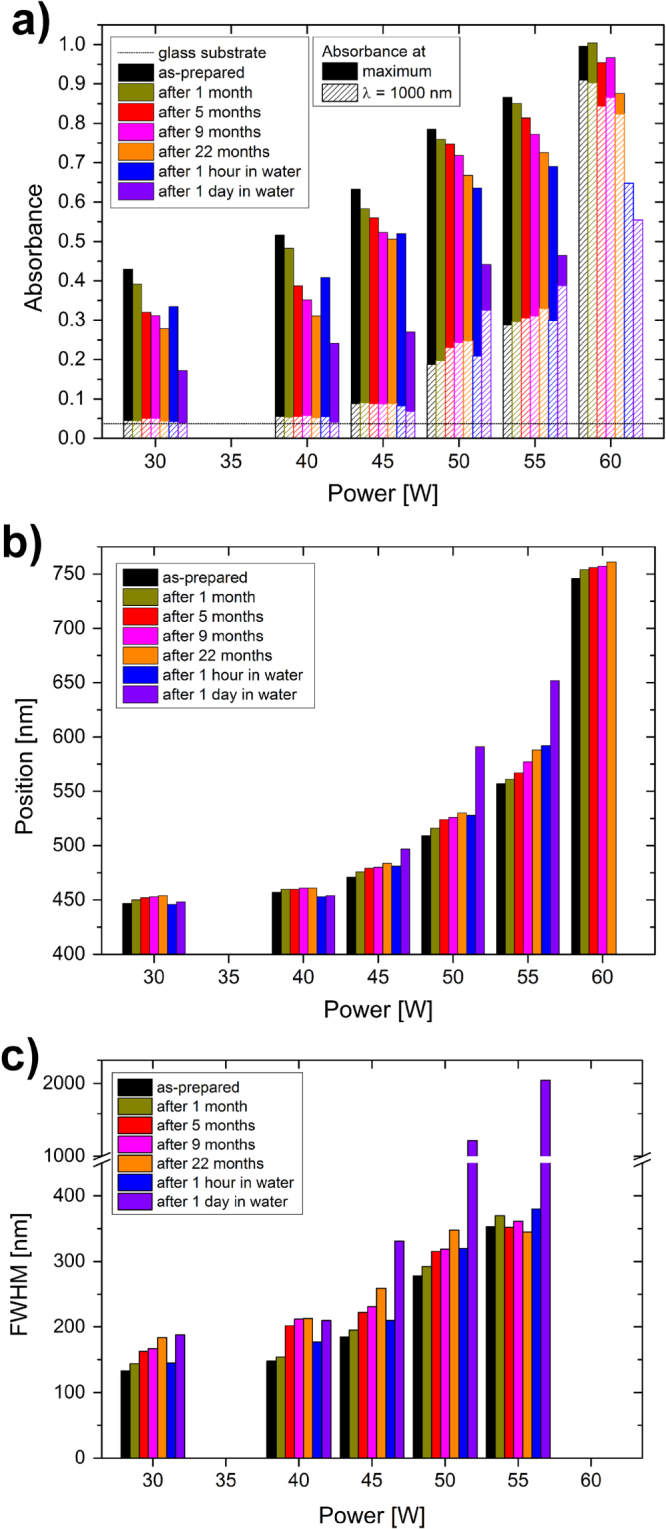
Changes in the parameters of the surface plasmon resonance of Ag/a-C:H:O nanocomposite films deposited at different RF powers during aging of the films in ambient air and in distilled water: (a) intensity of absorbance at the wavelength of the SPR and at the wavelength of 1000 nm, (b) position of the absorbance maximum, (c) width of the SPR peak (FWHM).

The systematic changes in the three SPR peak parameters can be observed in the following pictures. Particularly, the intensities of the absorbance are summarized in figure 9(a), their positions in figure 9(b) and the FWHM in figure 9(c). As for the absorbance intensity, the values were collected from two points of the spectra: at the position of the SPR maximum in the visible part of the spectrum and at the wavelength of 1000 nm. As was already reported above, the intensity of the SPR of the as-prepared films increases with the deposition power and decreases during aging. The overall decrease of the intensity is faster in water than in air and this rate increases with the higher filling factors of the films: for 30 and 40 W films, the absorbance intensity after aging for 1 h in water is at the level of about 5 months of aging in air, for the 45 W film, the aging for 1 h in water corresponds to about 9 months of aging in air, while the intensity after aging for 1 h in water is lower than after aging in air for about 22 months for the films deposited at 50 and 55 W. The absorbance maximum could not be localized in the case of the film deposited at 60 W because the peak is very broad and lies partly in the region where the noise level is higher due to the change of spectrometer detectors. Nevertheless, the overall decrease of the intensity of the absorbance peak is observed in all of the films. However, the situation is different when the changes in the NIR region are taken into account. The absorbance of the as-prepared films increases with the deposition power also in the NIR spectral range. Particularly, the intensity at the wavelength of 1000 nm increases from almost 0 in the case of films with low filling factors up to about 0.9 in the case of the film deposited at 60 W. The aging of the films however shows different effects. The level of absorption almost does not change (or very slightly decreases) in the case of films deposited up to the power of 45 W, while it increases in the case of the films deposited at 50 and 55 W. The intensity of absorption of the film with high filling factor decreases during aging in air or in water, while the decrease is much stronger in the latter case.

A gradual shift in the position of the SPR to higher wavelengths can be seen in the spectra of the as-prepared composites upon increasing the deposition power, as discussed above (figure 9(b)). Similarly, a gradual red-shift in the position of the SPR develops also during the aging of the films. However, the changes in the position of the peak induced by aging are not that strong as was previously observed in the case of the intensity of the peak. Nevertheless, the rates of the change are higher for the films deposited at higher powers for aging in air or water which corresponds to the changes observed in the case of the absorbance intensity. There are several microstructural parameters that affect the position of the SPR and will be further discussed in the next section.

The development similar to the position SPR can be observed also in FWHM of the absorbance peak (figure 9(c)). The values of FWHM of the absorbance peak in the spectrum of the as-prepared nanocomposite films increase with the increasing deposition power. The aging of the films in air or in water causes the broadening of the absorbance peak. Especially broad are the absorbance peaks in the cases of the films deposited at 50 and 55 W after their aging for 1 day in water. They resemble the spectrum of the film deposited at 60 W with well-percolated microstructure. It is interesting to notice that on the other hand, the changes in the width of the SPR peak of the 55 W film are only limited during aging in air.

To briefly summarize this section, we can say that the optical properties of the Ag/a-C:H:O nanocomposite films are modified during the aging of the coatings. Both media trigger qualitatively the same aging changes, particularly the decrease in the intensity of SPR, red-shift in the position of SPR and its broadening. However, similar to the case of microstructure, these changes are enhanced by the direct presence of water when compared to aging in air only.

### 3.4. Correlation between microstructure and optical properties of Ag/a-C:H:O films

It is well known that the optical properties of composites containing metal nanoparticles are closely connected to the characteristics of the respective material component and also microstructure of the composite [1, 56]. All of the microstructural (nanoparticle size, *d*_m_, size dispersion, *σ*, average shape factor, *S*_a_, permittivity of the matrix, *∊*_m_, and interaction between nanoparticles, NPI) as well as the optical (intensity, position, and FWHM of SPR) parameters are highly dependent on the deposition power, as could be seen in the discussion above or as reported elsewhere. This influence is summarized in table 1. As can be seen, all of them increase with the increasing power as more metal is incorporated into the plasma polymer matrix: more and larger particles (*d*_m_) with broader size distributions (*σ*) are formed which also increases the interaction between nanoparticles (NPI) due to decrease in the average inter-particle distances and thus enhancement of their dipole moments [58]. Permittivity of plasma polymer films (*∊*_m_) can be expected to increase with the increasing deposition power as was previously shown in other studies [17, 59]. The only exception is the average shape factor *S*_a_ which decreases instead due to the formation of island-like structures of highly irregular shapes at higher powers. On the other hand, the observed influence of aging on the film characteristics is more complex. The aging effect on some of the microstructural parameters, particularly *d*_m_, *σ*, and *S*_a_, and also on the position of SPR, is less pronounced in the case of the films with lower filling factors (can be regarded as constant) while it is more obvious for the films with higher filling factors (see figures 5 and 9). The border between the ‘low’ and the ‘high’ filling factor is not exactly drawn and differs from parameter to parameter. It is used here to stress that films with different filling factors might react to the aging with a different rate. Nevertheless, only the actual change in the respective parameter is recorded in table 1 for a better visualization.

**Table 1.** Observed influence of an increasing deposition power and aging on the microstructural and SPR parameters of the Ag/a-C:H:O nanocomposite films.

	Effect on microstructural parameter					Effect on SPR parameter		
	*d*_m_	*σ*	*S*_a_	*∊*_m_	NPI	Intensity	Position	FWHM
Increasing power	↑	↑	↓	↑?	↑	↑	↑	↑
Aging	↓	↓	↑	?	↓	↓	↑	↑

The effects of aging on the microstructure were found to be exactly opposite. All parameters decrease with the exception of *S*_a_: the loss of material through leaching of Ag^+^ ions results in decreasing of particle size, narrowing of size distribution and also decreasing the inter-particle interaction, while *S*_a_ increases as the island-like structure becomes separated into individual nanoparticles with more circular-like projections. The effect of aging on *∊*_m_ is not clear and requires an individual research to clarify the consequences. Nevertheless, it has been shown that *∊*_m_ of several plasma polymers increases in time due to water vapor penetrating the plasma polymer network [60].

The influence of the respective film microstructural parameters on the SPR is to a great extent interconnected, as can be seen in table 2. The nanoparticle size influences primarily the intensity of the absorbance maximum: the intensity increases with the increasing *d*_m_. On the other hand, increasing *d*_m_ causes a decrease in FWHM of the SPR peak which was found opposite when analyzing the spectra of the aging nanocomposites. Nevertheless, the dominant influence on FWHM has the size dispersion, *σ* [56], which correlates very well with the analysis of the spectra. The increasing shape factor *S*_a_ affects only position of SPR by shifting it to lower wavelengths. This was also observed on the spectra of the as-prepared films, however not in the case of aging of the films in air or in water. Nevertheless, the position of SPR is affected also by *∊*_m_ and NPI which correlate very well with the experiment. The permittivity of the matrix influences also the intensity of the absorption which is in opposite relation in the case of aging of the films (table 1). Nevertheless, the dominant effect on the intensity originates from *d*_m_. As can be seen, the optical properties (particularly the properties of SPR) of the as-prepared Ag/a-C:H:O nanocomposite films correlate very well with their microstructural parameters in the dependence on the deposition power. However, the influence of aging is a rather more complicated and a more precise comparison requires a modeling approach.

**Table 2.** Theoretical influence of increasing values of the respective microstructural parameters on the characteristics of SPR.

	Effect on SPR parameter		
Increase in microstructural parameter	Intensity	Position	FWHM
*d*_m_	↑		↓
*σ*			↑
*S*_a_		↓	
*∊*_m_	↑	↑	
NPI		↑	↑

## 4. Conclusion

Nanocomposite coatings of silver nanoparticles embedded in a plasma polymer matrix with different contents of metal can be deposited by simultaneous sputtering from a silver target and plasma polymerization of ethylene and carbon dioxide. The RF power supplied during the deposition has a major effect on the microstructure of the nanocomposite coatings. As shown by TEM, the size of the as-prepared nanoparticles, expressed via their equivalent diameter, increases from about 6 nm to about 60 nm when increasing the power from 30 to 60 W, i.e. small nanoparticles separated by the plasma polymer matrix grow larger and form a percolating island-like structure. In connection, also other microstructural parameters change. Particularly, the nanoparticle size dispersion, expressed in terms of standard deviation of the size distributions, broadens from about 2 nm for the films deposited at 30 W up to about 87 nm in the case of the films deposited at 60 W. On the other hand, the values of the shape factor of the projections of the nanoparticles decrease as particles with various sizes and shapes are deposited at higher powers. The optical properties of the nanocomposite films are closely connected to their microstructure. An anomalous absorption peak of the Ag nanoparticles is observed around 500 nm which is a typical feature of materials containing metal nanoparticles due to the effect of SPR. Similarly to microstructural parameters, also characteristics of the SPR peak depend on the deposition power. Particularly, the intensity of absorbance, the position of SPR, and the FWHM of the peak all increase with the increasing deposition power. It was observed that the as-prepared films are not stable, but rather undergo changes in their structure and optical properties when left for aging in ambient air or in distilled water. The aging processes in both environments are qualitatively the same. However, the processes are much faster and intense in water, where similar changes were induced on a time scale of hours as compared to months when aging in air. The aging of the nanocomposites is driven by water molecules that penetrate the coatings and induce changes in the structure and properties of the plasma polymer matrix as well as cause leaching of Ag^+^ ions from the surface of silver nanoparticles. The deposition conditions and the intrinsic microstructure of the as-prepared films affect the processes taking place during their aging. Three distinct aging regimes were observed. The formation of large silver crystals by Ostwald ripening was observed in the films with lower filling factors (below percolation threshold) deposited at lower powers. Such films with tuned microstructure with respect to Ag^+^ ion release might be suitable for application in biomedicine as antibacterial coatings. However, the growth of the crystallites needs to be taken into account and attention should be paid to the storage of the material prior to its use. Further, the films deposited at moderate power input possess relatively stable microstructure at the percolation threshold with a good balance of the size of the nanoparticles, the size distribution and the interparticle separation which makes them suitable for applications as various sensors. Finally, a degradation of the percolating island-like structure was observed in the films with high filling factors (above percolation threshold) deposited at higher powers. Nevertheless, these films could still be of interest for catalytic applications. In connection, the optical properties of the nanocomposite films are altered during their aging. The dominant feature in the optical spectra of the aging films is the decrease in their absorbance which is especially striking in the case of aging in water. This decrease is a direct consequence of the loss of silver material due to leaching of Ag^+^ ions.

The above discussed extremely thin films can be deposited within a short time and show some other advantages, e.g. they do not suffer from internal stresses and do not influence the mechanical properties of the substrate material. Nevertheless, it might be important to consider the requirements on the thickness of the films for any of their particular industrial applications.
